# Deposition and biokinetics of inhaled nanoparticles

**DOI:** 10.1186/1743-8977-7-2

**Published:** 2010-01-20

**Authors:** Marianne Geiser, Wolfgang G Kreyling

**Affiliations:** 1 Institute of Anatomy, University of Bern, Baltzerstrasse 2, CH-3000 Bern 9, Switzerland; 2 Comprehensive Pneumology Center, Institute of Lung Biology and Disease and Focus-Network Nanoparticles and Health, Helmholtz Center Munich, Munich, Germany; 3 German Research Center for Environmental Health, Ingolstaedter Landstrasse 1, D-85764 Neuherberg/Munich, Germany

## Abstract

Particle biokinetics is important in hazard identification and characterization of inhaled particles. Such studies intend to convert external to internal exposure or biologically effective dose, and may help to set limits in that way. Here we focus on the biokinetics of inhaled nanometer sized particles in comparison to micrometer sized ones.

The presented approach ranges from inhaled particle deposition probability and retention in the respiratory tract to biokinetics and clearance of particles out of the respiratory tract. Particle transport into the blood circulation (translocation), towards secondary target organs and tissues (accumulation), and out of the body (clearance) is considered. The macroscopically assessed amount of particles in the respiratory tract and secondary target organs provides dose estimates for toxicological studies on the level of the whole organism. Complementary, microscopic analyses at the individual particle level provide detailed information about which cells and subcellular components are the target of inhaled particles. These studies contribute to shed light on mechanisms and modes of action eventually leading to adverse health effects by inhaled nanoparticles.

We review current methods for macroscopic and microscopic analyses of particle deposition, retention and clearance. Existing macroscopic knowledge on particle biokinetics and microscopic views on particle organ interactions are discussed comparing nanometer and micrometer sized particles. We emphasize the importance for quantitative analyses and the use of particle doses derived from real world exposures.

## Introduction

It is expected that the use of nanomaterials and primarily of nanoparticulate materials will hold a key position in future science, technology and medicine. It spans from automotive, aircraft and space industry, to (bio)chemical and environmental engineering, to optics, electronics and communication technologies, to pharmacology and medicine, but also to day to day consumer products, i.e. to food, cosmetics and healthcare. The introduction of nanoparticulate materials is based on their exquisite properties and furthered by mandates of energy and resource savings. In this paper we will not focus on the manifold beneficial qualities of these new technologies but examine aspects of concern to pose risks for consumers and our societies. The exponentially increasing production of engineered nanoparticles urgently requires risk assessment of their potential for adverse health effects. Thereby, data for inhaled nanoparticles is particularly important because (i) this is the major route for unwanted exposure, and (ii) there is ample and consistent evidence for adverse health effects being associated with increased concentrations of ambient fine and ultrafine particles (e.g. [1-5]). Also, for nanoparticles with anticipated beneficial use, e.g. as diagnostic tools or therapeutics in medicine [6,7] health risk assessment is necessary. Even already tested materials have to be included since nanoparticles tend to have different properties compared to larger particles of the same material [8]. Risk assessment comprises exposure assessment, hazard identification and characterization, as well as risk characterization. The Scientific Committee on Emerging and Newly Identified Health Risks (SCENIHR) of the European Commission emphasized in its recent report on Risk Assessment of Products of Nanotechnologies [9] a number of critical factors for assessing the impact of nanoparticles and health: (i) so far, exposure assessment for existing nanomaterials is rudimentary. (ii) hazardous nanomaterials need to be characterized both "as manufactured" and in the various possible forms "as delivered" in bio- and eco-systems, (iii) the current lack of a generally applicable paradigm for nanomaterial hazard identification demands a case by case approach for the risk assessment of nanomaterials.

Studies in humans or large animal species are limited; hence we have to resort to rodents to obtain sufficiently detailed data for risk assessment by inhaled particles [10].

The focus of this paper is hazard identification and characterization, which starts with dosimetry, i.e. the evaluation of the deposition pattern of inhaled particles and their subsequent dissemination (biokinetics) in the respiratory tract and the entire organism. Therein microscopic analyses at the individual particle level provide detailed information about which cells and subcellular components are targeted by inhaled particles. Such studies intend to convert external exposure to internal exposure or biologically effective dose, and may help to set limits in that way.

We present and discuss primarily the results from studies we performed on the deposition, retention and clearance, as well as on secondary organ translocation of (insoluble) nanoparticles at a macroscopic and a microscopic level in view of contributing to nanoparticle characterization and identification of their potential risk. We compare data from inhaled nanoparticles (particles ≤ 100 nm in diameter) with those from micrometer-sized particles with diameters from 100 nm up to 10 μm. However, due to the blurred upper size limit of nanoparticles we consider micrometer-sized particles as those in the range of 0.5 - 10 μm. In addition, we include detailed methodological information allowing the application of protocols by the interested reader.

## Methods

### Methods for macroscopic studies

#### Quantitative biokinetics

**Figure 1:**
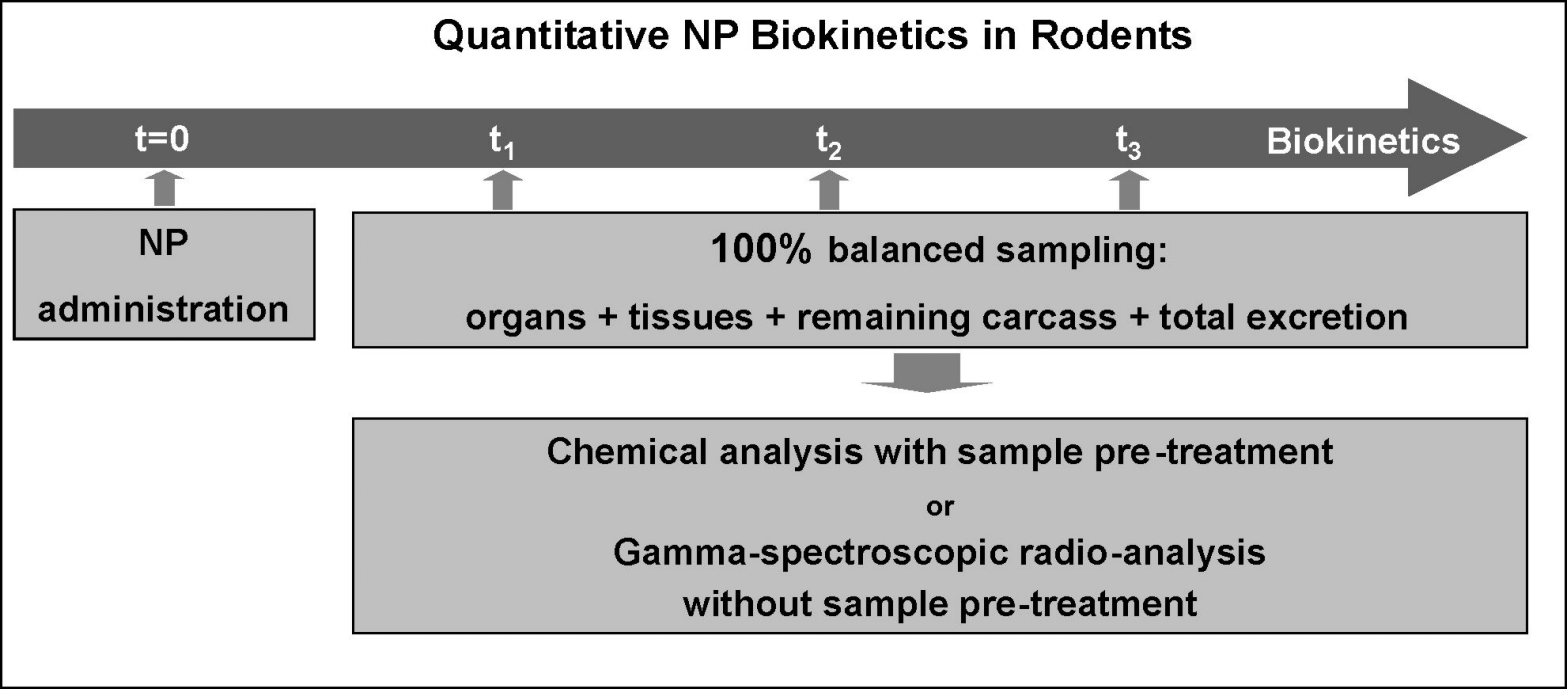
**Concept of quantitative nanoparticle biokinetics**. Nanoparticles (NP) are administered at time t = 0. From this time point on, the entire urinary and fecal excretions are collected separately. Animals are euthanized at times t_1_, t_2_, t_3_, etc., and organs and tissues of interest as well as the entire remaining carcass are sampled (100% balanced sampling). Samples require special preparation before chemical analysis, e.g. by inductive-coupled-plasma mass spectroscopy (ICP-MS). When nanoparticles are radio-labeled, samples will be analyzed directly, without any further preparation. Reprinted with permission from [21].

Quantitative biokinetics can be performed after nanoparticle administration by any route: by inhalation or intratracheal instillation to the respiratory tract, by gavage to the gastro-intestinal tract, by intravenous or intra-arterial injection to the blood circulation and by dermal applications to the skin. The concept is simple, aiming to estimate the total amount of nanoparticles in the entire body at a certain time point after exposure and in all excretions until this time point. Hence, not only the distribution of nanoparticles in organs and tissues of interest is assessed, but nanoparticles in the remaining carcass and those excreted are also included. With this, a 100% balance to the administered nanoparticles is achieved and a complete, yet detailed, quantitative analysis of their biokinetics is obtained [11,12]. For this purpose, particle distribution is measured at several time points after particle exposure, as shown in Figure 1. Although this approach is applicable to any animal species, ethical, economic and practical reasons restrict quantitative biokinetics to small laboratory animals.

Analytical chemistry e.g. inductively coupled plasma mass spectroscopy (ICP-MS) or atomic absorption mass spectroscopy (AA-MS) provides suitable methods to quantify particles in the collected specimens. Thereby, special attention has to be paid to sample preparation, i.e. when nanoparticles need to be dissolved, the extremely low content of ions resulting from the very low nanoparticle mass may lead to substantial ion loss to the walls of the reaction vessels prior to sample analysis. Radio-spectroscopy may provide an elegant alternative, allowing direct analysis of native organs and tissues without any pre-treatment. In this case, the radio-label needs to be firmly integrated into the nanoparticle matrix without any leaching. While this requirement is hard to fulfill, when a radio-isotope is blended into the nanoparticles during production, stable labeling is obtained, when the radio-isotope belongs to the same chemical element as the nanoparticle matrix. The latter, requiring integration of the radio-label during nanoparticle production, however, is often difficult, since nanotechnology laboratories are usually not equipped with radio-chemistry instrumentation and the necessary permission. Nuclear reaction within the beforehand generated unlabeled nanoparticles upon neutron, proton or any other ion bombardment, allows radio-activation of one to a few atoms of the nanoparticles. Mostly, the amount of radioactivity is sufficiently high for subsequent radio-analysis, when a single atom per nanoparticle was converted by the nuclear reaction. A well known example is gold that is neutron-activated in a nuclear research reactor such that the gold nanoparticles are labeled with the ^198^Au radio-isotope.

#### Animals and organ preparation

For our studies with radio-labeled nanoparticles [12,13], the deeply anesthetized (isoflurane 5%) rats were killed by exsanguinations via the prepared abdominal aorta. Like this, about 70% of the total blood volume, estimated from the body weight, was collected. All organs and tissues, the remaining carcass as well as the total excretions were thereafter sampled for radio-analysis:

- Organs: lungs, liver, spleen, kidneys, reproductive organs, brain, heart, gastro-intestinal tract, blood, skin

- Tissues: samples of muscle and of bone (femur)

- Remainder: carcass beyond the listed tissues and organs

- Excretions: urine and feces, collected separately

To avoid any cross contamination, the organs were collected *in toto *and all body fluids were immediately removed, when vessels or excretory ducts had to be cut. Organs and tissue samples as well as the entire excretion were collected such that the entire organism was sampled and weighed in wet state.

The application of clean dissection techniques is essential to avoid cross contamination, in particular in inhalation studies, where fur contamination occurs upon whole body or nose-only exposures. Systematic changes of dissection tools and equipment are highly recommended. In addition, whole body vascular perfusion to empty the blood vessels within the organs is recommended to estimate particle retention in the parenchyma.

### Methods for microscopic studies

Analysis of nanoparticles at the individual particle level by electron microscopy requires (i) adequate organ preservation, (ii) representative tissue sampling, and (iii) unambiguous identification of the nanoparticles in ultrathin tissue sections.

#### Lung fixation

The methods for tissue preservation using chemical fixative solutions are well established. The following methods are generally used for whole lung fixation:

##### Airway instillation

The fixative is introduced into the airways of a collapsed lung, in deeply anaesthetized animals or in cadaver lungs [14]. Thereby, the air spaces evenly expand and the interalveolar septa remain unfolded. *Note*: Airway instillation of (aqueous) fixatives does not preserve the lung lining layer and luminal cells, i.e. macrophages are dislocated from their native positions [15].

##### Vascular perfusion

Fixatives are delivered to the lungs via the blood vessels, while the airways and alveoli remain in their natural air-filled state [14,16]. This method is less effective in larger airways and especially of large animals, due to larger distance between vasculature and airway surface. *Note*: Fixation solely with glutaraldehyde, paraformaldehyde, or mixtures thereof, does not adequately preserve the lung lining layer and its associated luminal cells. Osmium tetroxide and uranyl acetate are necessary for cross-linking and stabilizing this layer [17,18].

##### Other methods

**Figure 2:**
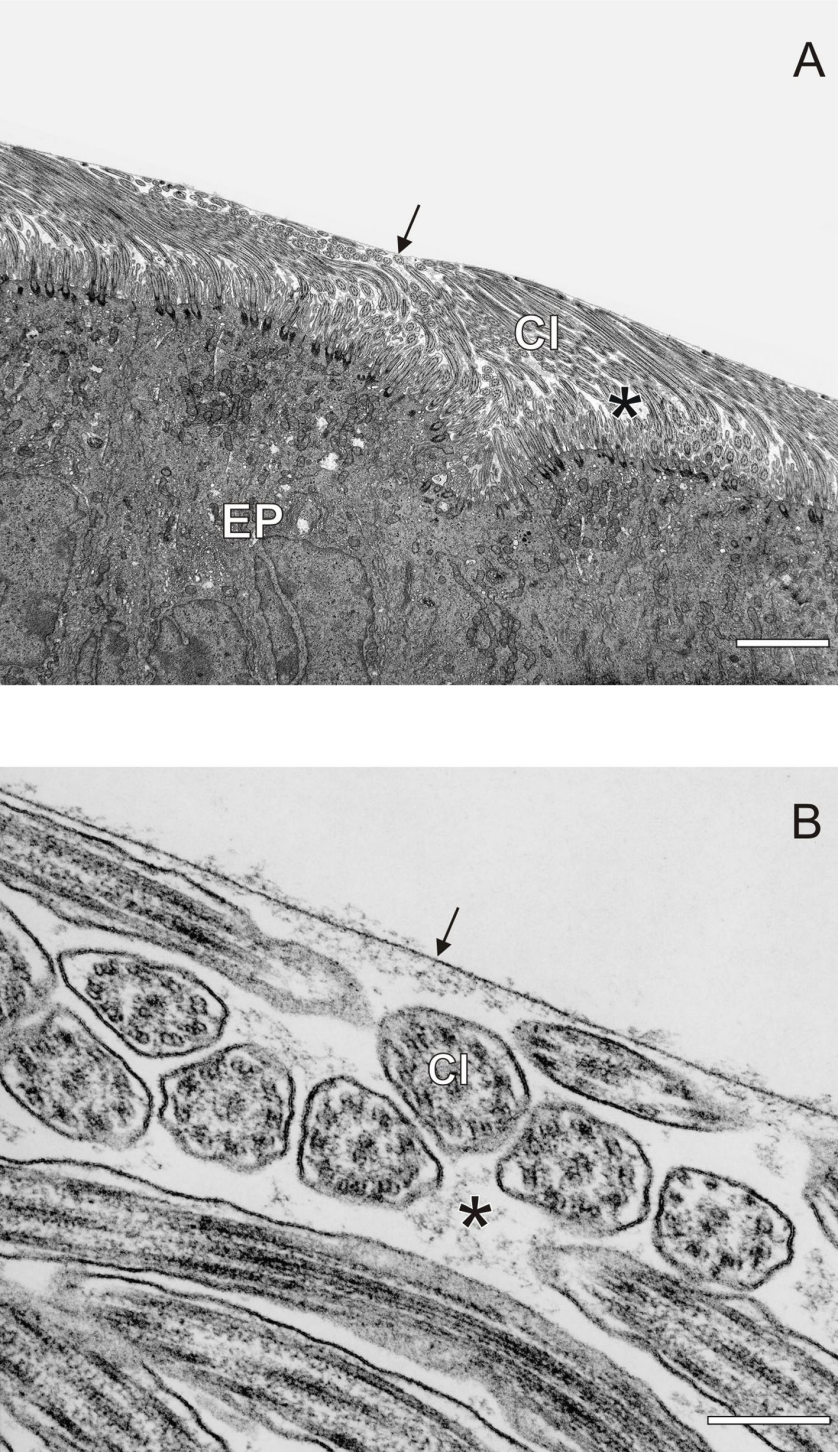
**Micrographs of horse trachea fixed by immersion in non-polar fixative (A, B)**. Note that the lung lining layer (aqueous phase [asterix] and surfactant film at the air liquid interface [arrow]), has been preserved with this fixation technique. EP = epithelium, CI = cilia. Bars: (A) = 2 μm, (B) = 0.2 μm. Reprinted with permission from [21].

Vapor fixation of lungs at a given pressure is a fast and technically easy method to preserve whole lungs (e.g. [19,20]). However, shrinkage and organ distortion occur and ultrastructural preservation of cells is inferior. Non-polar fixatives, i.e. 1% osmium tetroxide dissolved in inert fluorocarbon (FC, Fluorinert™ Liquid, 3 M, Belgium) are suitable to preserve large airways, either by immersion of excised specimens (Figure 2, [21]) or by airway instillation [17,22,23]. A variety of other fixation techniques have been developed in view to preserve the inner surface of airways and alveoli, but most of them cannot be used to fix whole lungs or even lung lobes (for review, see e.g. [17]).

#### Recovery of phagocytic cells by bronchoalveolar lavage

Particle uptake by airway and alveolar macrophages can be studied in two ways: (i) by fixing whole lungs or parts thereof to study the cells in situ, within their functional environment [24-26]; the cells' location on surfaces requiring special fixation protocols (see above); (ii) by isolating the cells from the organ and further processing for microscopic analysis; the lung surface cells being readily accessible to bronchoalveolar lavage (BAL).

In small laboratory animals, surface cells are usually recovered by whole lung lavage. In humans, BAL cells are recovered from the lower respiratory tract (bronchi, bronchioli and alveoli) using flexible fiberoptic bronchoscopy. The standard site of sampling is generally the middle lobe or lingula. *Note*: Not all macrophages will be recovered by BAL, and it is not known, whether these macrophages form a distinct population [27].

#### Morphological characterization and elemental microanalysis of nanoparticles

**Figure 3:**
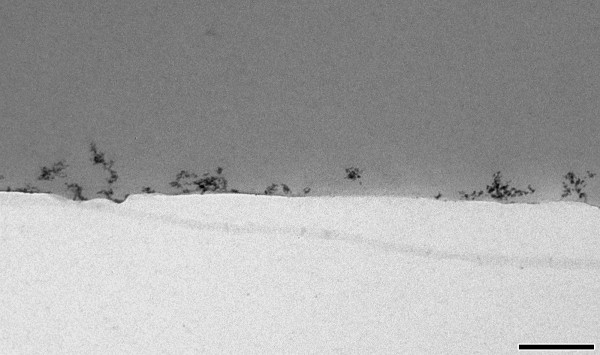
**Nanoparticle morphology**. Section profiles (transects) of TiO_2 _nanoparticles collected from the aerosol on filters, embedded in Epon and cut perpendicularly to the filter. Note that the as 20 nm measured TiO_2 _aerosol particles are already agglomerates of smaller primary particle structures of 3 - 5 nm formed immediately after spark ignition and condensation. Bar: 200 nm.

Unambiguous identification of nanoparticles in ultrathin tissue sections is a prerequisite and requires in most cases their morphologic classification as well as elemental microanalysis. Particle morphology is established (i) on aerosol samples collected directly on formvar coated tsansmission electron microscopy (TEM) grids using electrostatic precipitation and (ii) on ultrathin sections of aerosol samples collected on filters (Figure 3). Like this, the morphology of nanoparticles in 3 d as well as in 2 d (transects) is established.

**Figure 4:**
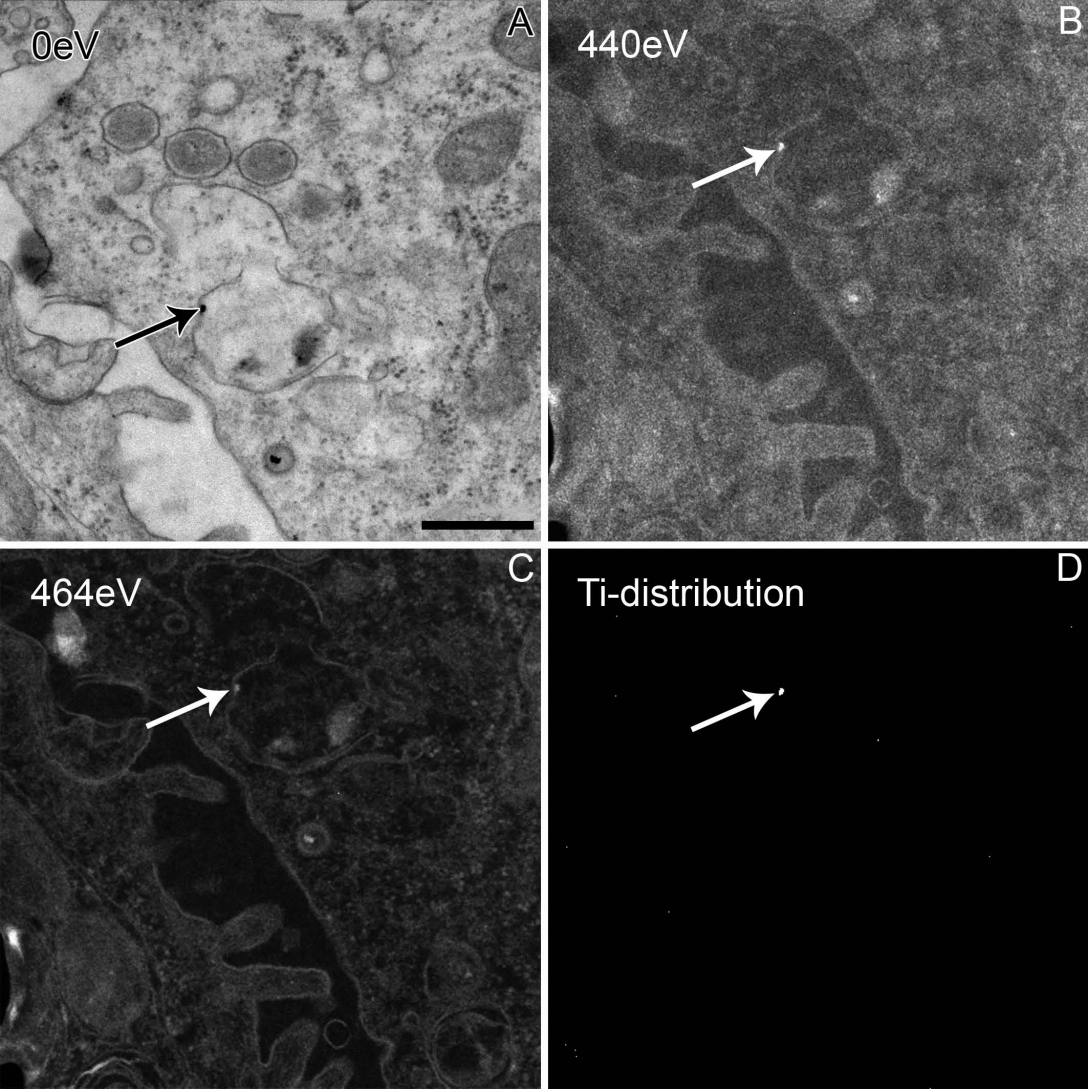
**Elemental microanalysis of a particle in lung tissue by electron spectroscopic imaging (ESI, three window method), demonstrating that the nanoparticles (arrows) consists of titanium, adapted from **[21]. The first image (A), taken at 0 eV, shows the structural details; it is slightly shifted as compared to the images used for elemental microanalyses (B-D). For elemental microanalysis, three images are taken: two below the element-specific edge in order to extrapolate a background image, at ΔE = 390eV (not shown) and ΔE = 440eV (B), respectively, and one image within the maximum of the element specific signal at ΔE = 464eV (L_2,3 _edge of titanium) (C). The net titanium signal (D) is calculated by subtraction of the extrapolated background image from the titanium specific signal. ESI images have reversed contrast as solely inelastically scattered electrons are used with an energy loss producing a dark field image. Hence, the obtained image reflects the titanium distribution in white pixels. Bar: 500 nm.

Because there are other structures in the ultrathin tissue section that resemble the particles morphologically ("false positives"), additional elemental microanalysis of the nanoparticles by energy filtering transmission electron microscopy (EFTEM) (e.g. LEO 912, Zeiss, Oberkochen, Germany) is necessary. We have adapted three high resolution and sensitivity methods for elemental analysis of TiO_2 _nanoparticles in ultrathin sections [28], from which we routinely use electron spectroscopic imaging (ESI), where images are taken at a defined energy loss. Elemental (titanium) mapping is achieved with the three window method (Figure 4).

#### Systematic uniform random tissue sampling and particle quantification

**Figure 5:**
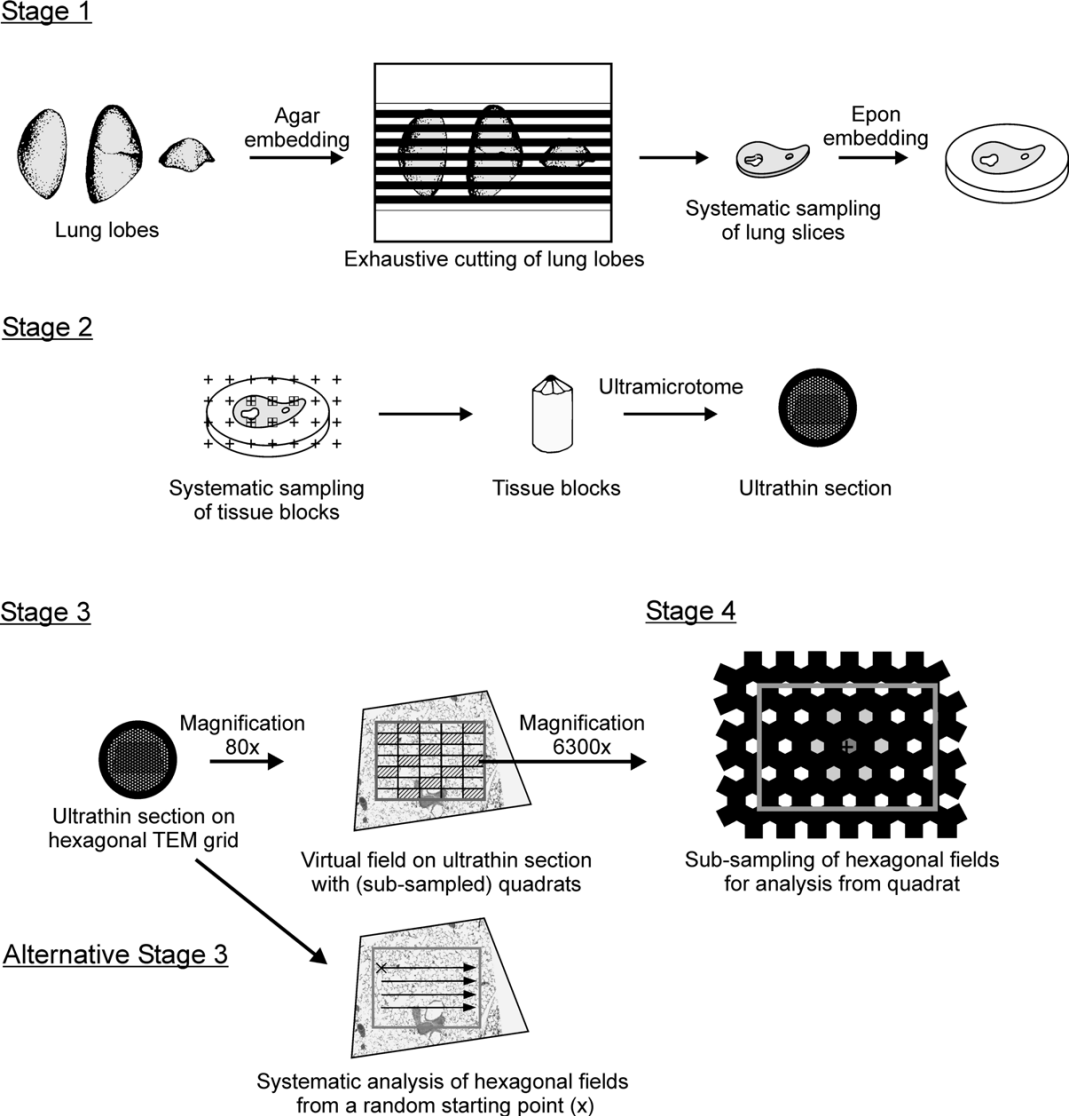
**Multistage tissue sampling design for EFTEM analysis of nanoparticles, adapted from **[21]. *Stage 1 - Lung slices*: exhaustive cutting of agar embedded lung lobes (with random start) into equally thick slices [38], followed by systematic sampling of slices, e.g. every second (with random start) and Epon embedding. *Stage 2 - Tissue blocks and ultrathin sections*: systematic sampling (with random start) of tissue blocks from lung slices using a point counting test system and cutting of ultrathin (≤ 50 nm) sections, which are placed on 600-mesh hexagonal copper grids and stained with lead citrate and uranyl acetate. *Stage 3 - Quadrats on ultrathin sections*: generation of a virtual field, completely contained within the ultrathin section, at 80× magnification. Field subdivision into a predetermined number of uniform quadrats and systematic subsampling of quadrats thereof (marked in grey). *Stage 4 - Fields for nanoparticle analysis*: Subsampling of a group of seven adjacent fields, delimited by the hexagonal TEM grid bars, within each quadrat at 6300× magnification, using a point counting test system. Tissue analysis within these hexagonal fields for (i) the presence of particles with matching nanoparticle characteristics and (ii) particle localization within the compartments of interest. Stages 3 and 4 can equally be applied on ultrathin sections of cell pellets. *Alternative to stages 3 and 4 *- Fields for nanoparticle analysis are sampled by picking a random hexagonal field on the ultrathin section as starting point at 80× magnification. From there on systematic tissue analysis in horizontal and vertical direction, using the automated goniometer of the microscope.

To investigate the spatial and temporal distribution of particles in cells, tissues and organs quantitatively, specimen selection must confine to some randomness, i.e. ensure that every part of the organ and every orientation of a structure or particle therein have the same chance of being selected [29]. Systematic uniform random sampling, where the first item is selected at random position and orientation and the following ones in a predetermined interval, is most efficient and is superior to random sampling. Specimens for microscopic analysis are usually sampled in a hierarchical mode, called multistage or cascade sampling [30]. We have developed the 4-stage sampling protocol shown in Figure 5 for electron microscopic analysis of nanoparticles in lungs [26,31]. The last sample consists of fields, delimited by the bars of the hexagonal TEM grids, sampled on ultrathin sections of tissue or cell pellets. These hexagonal fields are then analyzed for the presence of particles with matching morphology and material properties. In case of inhaled 20 nm TiO_2_, the particles have to correspond to transects of the aerosol particles and to consist of titanium upon elemental microanalysis [26,31,28]. Next, the nanoparticles are assigned to the compartments of interest.

Recent methods for quantifying nanoparticles using TEM sections have been reviewed in detail by Mayhew and colleagues [32]. Therein, especially the methods for relative quantification of nanoparticles allowing between-group and within-group between-compartment comparisons are readily applicable and have been used already [e.g. [31,33,34]]. They are usually performed using particle transect counts on ultrathin sections. Methods for absolute quantification counts are performed in 3-dimensional space, using pairs of physical sections, the physical disector, or pairs of optical sections in a thick section, the optical disector (e.g. [35-37]). We have implemented this technique to count micrometer sized particles and airway macrophages in lungs by light microscopy (e.g. [25,38]). The use of (physical or optical) disectors for total number estimates of inhaled nanoparticles, however, is currently not practicable in electron microscopy, foremost because of the high magnification required for particle identification, but also because of the low number of particles that penetrate into the lung tissue [26]. The latter is also the reason why electron tomography that allows the application of the advanced stereological tools described above can currently not be used in realistic inhalation studies.

## Particle deposition and biokinetics

### Particle deposition and lung anatomy

**Figure 6:**
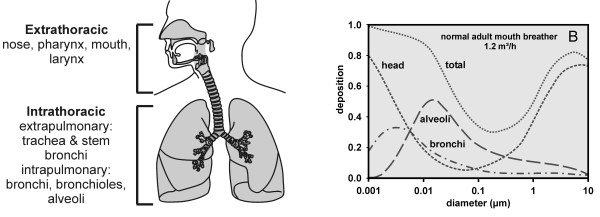
**The respiratory tract (A) and particle deposition in a normal adult mouth breathing male human subject at rest, as a function of particle size (B)**. Data of bronchi are the sum of the deposition in bronchi and bronchioles. Adapted from [105] and [39] and reprinted with permission from [21].

The mechanisms, the pattern and the efficiency of particle deposition in the respiratory tract largely depend on the aerodynamic or thermodynamic diameter of the inhaled particles (Figure 6). Nanoparticles deposit with high efficiency in the entire respiratory tract, from the head airways to the alveoli, due to diffusion. Therefore, only their thermodynamic diameter is relevant during inhalation; the aerodynamic diameter remains irrelevant, because sufficiently strong drag forces are absent.

The Human Respiratory Tract Model (HRTM) of the International Commission of Radiological Protection (ICRP) provides deposition data of inhaled particles from 1 nm to 10 μm of healthy adult female and male human subjects, at different breathing patterns and physiological activities [39]. Data are given for the extrathoracic, the bronchiolar (i.e. sum of bronchi and bronchioles) and the alveolar regions. These data, which we recently discussed [40,41], represent a meta-analysis of the knowledge at the time of their publication; they are widely accepted and used in many applications. The deposition data given for children, however, were solely derived by applying numerical scaling factors to the data from adults. In fact, there is considerable lack of knowledge on the deposition in infants and children. Furthermore, there is ample evidence for significantly altered deposition of nanoparticles in patients with lung disease, due to changes in the breathing pattern and of fine pulmonary structures (for review [40-42]).

**Figure 7:**
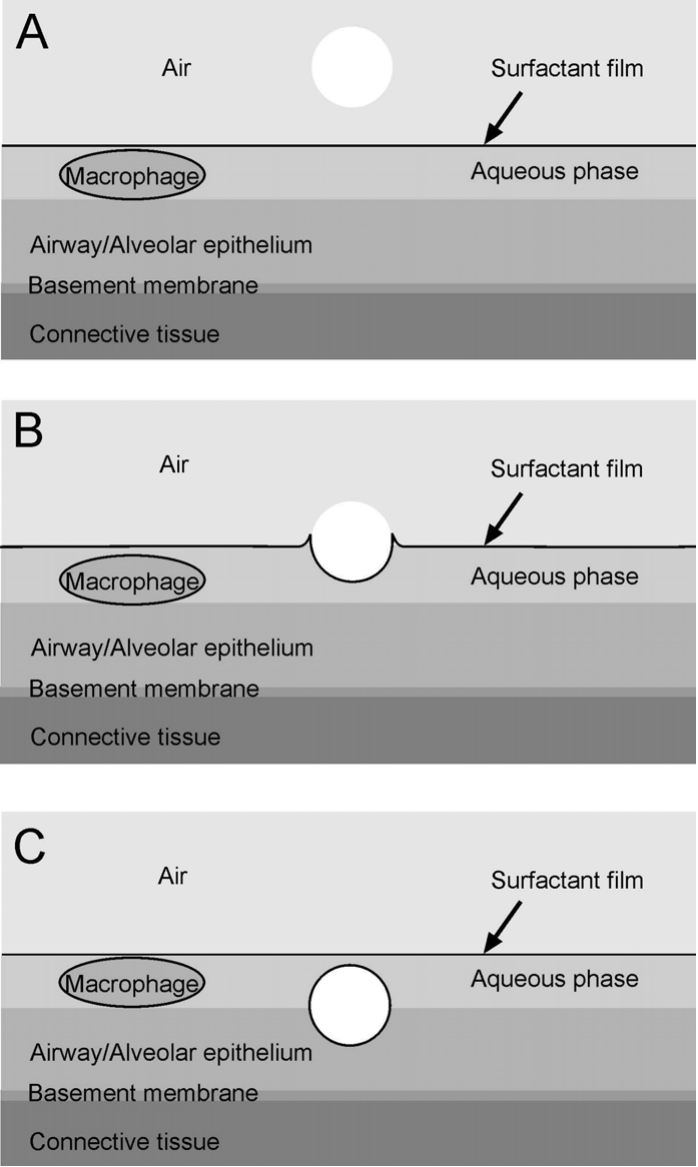
**Components of the inner surface of the lungs (A); particle deposition and immediate wetting (B) and complete displacement (C) of the deposited particle (white sphere) into the lung lining layer by surfactant**. Reprinted with permission from [21].

Despite considerable variations in airway and alveolar size and cellular composition,n as well as substantial interspecies differences, the airway and alveolar walls are built of the same basic structural elements (Figure 7A). In view of the inhaled particle these are: (i) the liquid lining layer consisting of the surfactant film at the air-liquid interface and the aqueous phase (periciliary layer and mucus) beneath it, (ii) the mobile cells, i.e. mainly resident airway and alveolar macrophages submersed in the aqueous phase; (iii) the highly differentiated epithelium with its basement membrane and (iv) the subepithelial connective tissue containing the blood and lymphatic vessels, and further cells of the immune system. The inner surface of the lungs functions as a physical, biochemical and immunological barrier, separating the outside from inside. It is also these structures deposited particles first interact with.

### Particle retention and relocation pathways within the lungs

The retention of particles starts with their wetting and subsequent displacement from the air into the aqueous phase by surfactant (Figures 7B/C); regardless of shape, surface topography and surface free energy [43-46]. Though experimentally demonstrated yet for microparticles of 1 - 6 μm in diameter and fibers, the same can be expected for nanoparticles, since this process becomes even more efficient with decreasing particle size.

In general, microparticles (0.5 - 10 μm, see our definition in the Introduction) remain on the epithelial surface in airways and alveoli and are accessible to BAL, as experimentally best demonstrated in rodents [47,48]. Their retention time depends on the deposition site and on the interaction of the particles with the inner lung surface. It is short for particles deposited in conducting airways due to efficient mucociliary and cough clearance, but it increases with airway generation number, as a consequence of increasing pathway length and decreasing mucus velocity. While the retention of microparticles generally does not exceed 24 - 48 h in rodent airways, there is evidence for prolonged retention of microparticles in airways of dogs [49] and of both micro- and nanoparticles in small airways of humans [50,51], the probability being inversely correlated to particle size. In the latter studies, a maximum of long term retention of up to 80% was found for particles ≤ 100 nm. Further evidence for a correlation between geometric particle diameter and prolonged particle retention in airways was recently obtained from a study targeting 100 nm carbon particles to human airways by shallow aerosol bolus inhalation [42,51]. In this study only 25% of the nanoparticles were removed by mucociliary clearance within 24 h, while 75% were retained for more than 48 h. Possible explanations for these findings are that the particles were no longer accessible to mucociliary clearance either because they penetrated through the mucus deep into the periciliary phase [43,46], or that they were deposited in areas with reduced lung lining layer. In both cases, further interaction of particles with cells of the inner lung surface, i.e. macrophages, dendritic and epithelial cells is furthered [52], and the probability for particle relocation beyond the epithelial barrier enhanced.

Well known and recognized is the interaction of particles with lung surface macrophages. As we discuss in detail in the subsequent section, phagocytic uptake of particles is a key factor for particle clearance in airways and alveoli, also contributing to particle retention. Evidence for relocation of microparticles to interstitial sites and long term retention was obtained from biokinetics and subsequent morphologic studies in dogs [50,53]. There are only few in vivo data of nanoparticles retained within the lung tissue. We found 20 nm TiO_2 _nanoparticles, though a small fraction, to penetrate into epithelial cells and deeper into the lung tissue within only 1 h after aerosol inhalation in rats [31]. From this electron microscopic study, there was no evidence for substantial accumulation of nanoparticles in the lung tissue within 24 h post aerosol inhalation. Evidence for considerable particle relocation into the pulmonary tissue over a longer period of time, however, was obtained in a six month study in rats that had inhaled 20 nm iridium particles during 1 h [12,13]. In this study, as shown in Table 1, 46% of the nanoparticles were accessible to exhaustive BAL immediately after aerosol inhalation; most of them (78%) were not associated with macrophages. At 24 h and later, only about 10% of the nanoparticles were accessible to BAL, and from 72 h on most of them (90%) were associated with macrophages. It is not yet known by how much the material determines the extent of particle relocation into cells and beyond epithelial barriers. For clarification of the observed differences between TiO_2 _and iridium nanoparticles described above, additional studies of iridium nanoparticles at the individual particle level, i.e. by (EF)TEM, and of the biokinetics of adequately labeled TiO_2 _nanoparticles are required.

**Table 1:** Recovery of inhaled iridium nanoparticles by BAL and association of nanoparticles with BAL macrophages

Time of BAL after aerosol inhalation (h)	Particle recovery by BAL (% lung retention)	Particles associated with BAL macrophages (% lavaged nanoparticles)
0	46	22
6	19	62
24	11	70
72	10	92
168	9	97

As a result of the relocation of particles from the lung surface into the pulmonary tissue, such particles may become accessible to lymphatic drainage and they may enter blood vessels favoring subsequent dissemination into secondary organs. Surprisingly, a rather small fraction of microparticles retained in the interstitial lung tissue in dogs was cleared to the hilar lymph nodes, and there was no detectable particle translocation into the blood circulation during the entire retention period [50,53]. Though not studied, macrophage mediated re-appearance of microparticles on the lung surface for subsequent transport towards the larynx was suggested, as more than 90% of the microparticles were found to be associated with BAL macrophages at any time after inhalation. In our 24 h study with TiO_2 _nanoparticles in rats lymphatic drainage was not assessed and very few particles were found to have penetrated into the blood vessels [31]. Furthermore, in the above mentioned study with iridium in rats [12,13] there was no noticeable accumulation of nanoparticles in tracheal lymph nodes and little translocation into the blood, as altogether about 10% of the deposited nanoparticles were found to be retained in all secondary target organs, the skeleton and soft tissue [54]. Instead, and as observed for microparticles retained in the interstitium of dog lungs, the long term retained iridium nanoparticles apparently re-appeared on the epithelium. And again, subsequent macrophage mediated clearance to the larynx is likely, since more than 90% of the iridium nanoparticles were associated with BAL macrophages at and beyond 72 h after aerosol inhalation.

### Particle transport towards the larynx

#### Conducting airways

The major pathway for particle clearance from conducting airways is by mucociliary transport. The transport rate depends on both the cilia and the lung lining layer; it is fastest in the central airways and gets slower with increasing airway generation (for review [50]).

In rodents, microparticles in airways are usually cleared within 24 - 48 h and the clearance of nanoparticles seems to be fast as well. Kinetic studies with iridium nanoparticles revealed a clearance of about 30% of the deposited particles within the first 24 h after aerosol inhalation (rat [11], mouse [55]). The experimental data in rats correspond rather well with theoretical ones [56]; free public software "Multipath Model of Particle Deposition, MPPD" is available for particle deposition and clearance estimates in humans and rats http://www.thehamner.org/mppd/helpfiles/index.htm. However, there is evidence that mucociliary clearance may not remove all particles from the lung surface, particularly in dogs and humans, resulting in prolonged particle retention, as we discussed in the previous section. These apparent differences between species have to be utterly considered when extrapolating rodent data to man. As mentioned also in the previous section, the mechanisms for prolonged retention and, hence, slower clearance of particles from airways remain unclear. Yet, prolonged retention favors accumulation of inhaled material within the tissue, i.e. leads to increased lung burden, which may be a factor for small airway cancer development. In fact, the HRTM model of the ICRP already included the element of prolonged airway retention.

Resident macrophages also contribute to the clearance of particles from conducting airways. Data from studies in hamsters with particles of 3 - 6 μm in diameter and of different materials, showed an average uptake of 28% (SD 16%) of particles deposited in airways by macrophages within less than 1 h after aerosol inhalation and of more than 80% of the remaining particles at 24 h [24]. From this study there was also evidence for rapid (mucociliary) clearance of macrophages with high particle loads.

#### Respiratory bronchioles and alveoli

Particle uptake by resident surface macrophages and further transport to the larynx is the predominant mechanism for particle clearance from the peripheral lungs. As in the conducting airways, the internalization of microparticles is rapid and essentially complete within 24 h [25,57,58].

The transport rate of microparticles, i.e. their clearance from the lungs varies between species; it is one order of magnitude lower in man, monkey, dog and guinea pigs than in rodents and sheep [50,59]. One anatomical feature of the lung, i.e. the number of generations of respiratory bronchioles correlates rather well with these observed species specific differences: rat, mouse, hamster and sheep have 0 - 2 generations of respiratory bronchioles, whereas man, monkey and dog have 3 - 5 [60]. There is one exception: the particle clearance rate is slow in guinea pigs, although they have only about one generation of respiratory bronchioles. Species specific differences in particle relocation from the surface into the lung tissue, as discussed in the previous section, may also contribute to the observed variations. Respective correlations can be deduced from experimental studies with microparticles, which are transported to the interstitium in human or canine lungs, but remain on the epithelium in rodent lungs (hamsters: [48,61]).

Effective surface macrophage mediated clearance rates have been found to be the same for micro- and nanoparticles in rodent lungs [13]. Hence, one could extrapolate from both lines of evidence that in human beings nanoparticles penetrate the lung epithelium for long term interstitial retention like the microparticles and consequently the clearance kinetics of nanoparticles is as slow as that of microparticles. The underlying mechanisms are not yet fully understood and presumably more complex. Note, however, that due to the very slow particle clearance kinetics in humans, with declining particle clearance rates over increasing retention time, an estimated fraction of 10 - 20% of insoluble particles will never be cleared out of the human lungs under physiological conditions [50]. In cases of very high particle exposure, like in smoking or in some occupational settings (mining, milling, etc.), the fraction of never-cleared particles may be substantially enhanced and associated with fibrotic pathogenesis.

**Figure 8:**
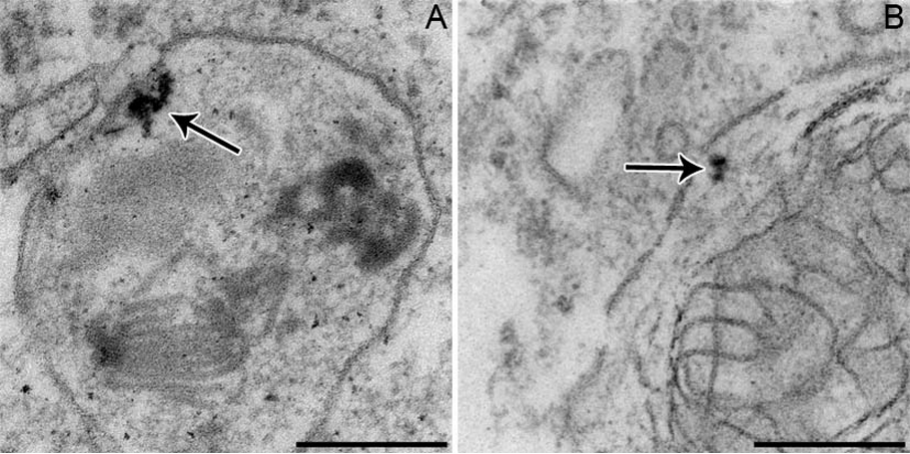
**TEM micrographs (A) and (B) of inhaled TiO_2 _nanoparticles (arrows) located in large phagolysosomes, which contain other phagocytosed material, in mouse surface macrophages**. Bars: 200 nm.

We have recently assessed the clearance of inhaled 20 nm TiO_2 _particles by surface macrophages at the individual particle level by EFTEM [26]. The data from this study in rats showed that surface macrophages do not efficiently phagocytose these nanoparticles but take them up rather sporadic and non-specific within the first 24 h after particle inhalation: (i) There was only about 0.1% of the TiO_2 _nanoparticles internalized by macrophages within 24 h after aerosol inhalation, compared to > 10% of microparticles that were phagocytosed already within 1 h [24] and > 80% within 24 h after aerosol inhalation [38,57,58,62,63]. (ii) As little as 0.2% and 1.7% of the BAL macrophage populations contained nanoparticles at 1 h and at 24 h, respectively, after the aerosol inhalation, which is about two orders of magnitudes less than what was shown for 3 - 6 μm particles of different materials [24,25]. (iii) The TiO_2 _nanoparticles in BAL macrophages were not tightly enclosed by the vesicular membrane, as it is known from phagocytic uptake of microparticles. Instead, nanoparticles were located in large vesicles that mostly contained other material (Figure 8). This also points to a rather sporadic uptake of TiO_2 _nanoparticles by surface macrophages, maybe during the process of phagocytic uptake of other material. Hence, there is evidence from these studies that, at least within the first 24 h after aerosol inhalation, nanoparticles bypass the most important clearance mechanisms for particles deposited in the alveoli, namely phagocytic uptake by surface macrophages. Consequently, the probability of uptake by epithelial cells and/or relocation through the thin epithelial barrier increases for nanoparticles.

Despite all the variations discussed above, the main pathway for particle clearance in airways and alveoli, for free and phagocytosed particles, for micro- and nanoparticles, is towards the larynx. Even particles that were relocated into the underlying interstitium seem to reappear again on the lung surface to be cleared this way.

### Other particle clearance pathways

#### Particle clearance via the lymphatic system

Particle clearance through lymphatic drainage may be expected in lungs where significant particle retention in the interstitial space has been shown, i.e. microparticles in dogs and iridium nanoparticles in rats. However, in dogs only a small, intersubject variable fraction of 1 - 5% of deposited microparticles was found to be drained to the regional lymph nodes under physiological conditions [50,64]. Though there are no human long term lymphatic clearance data available, we expect essentially the same fate for microparticles as in dog lungs, because of the striking similarities of particle clearance kinetics in these two species. There was also no noticeable iridium nanoparticles accumulation found in tracheal lymph nodes of rats [12,13].

Still unclear is the role of bronchus associated lymphatic tissue (BALT) by which particles would find their way back to the airway epithelium, via particle drainage into the lymphatic vessels and the blood circulation, as it was hypothesized many years ago [65-67].

#### Particle transport from the interstitium onto the epithelial surface

As discussed above, there is evidence for re-appearance of microparticles on the lung epithelium in dogs and humans, and of nanoparticles in rodents, because the most prominent clearance pathways of long term (in the interstitium) retained particles were shown to be surface macrophage mediated and directed towards the larynx (dogs [53], rats [13]). This clearance pathway is obsolete in rodents, which have negligible interstitial retention of microparticles [48,61]. So far there are no human or large species data available on the long term retention and clearance of insoluble nanoparticles from the peripheral lungs. The re-appearance of nanoparticles on the lung epithelium from the interstitium is likely to be macrophage mediated. As discussed above, we cannot yet exclude that particles may also re-appear on the lung epithelium as a consequence of their release into the lymphatic vessels from BALT [65-67].

#### Particle translocation to the blood and accumulation in secondary target organs

For more than a decade, epidemiological studies have indicated associations between exposure to increased concentrations of ambient fine and ultrafine particles and adverse health effects in susceptible individuals [1,4,5,68]. Cardiovascular effects observed in these studies triggered the discussion on increased transport of inhaled ultrafine particles from the epithelial surface towards the blood circulation and subsequently to target organs, like the heart, liver and brain, eventually adversely affecting the functions of these organs [69].

From the latter review, nanoparticles are apparently transported across membranes with hardly any restraint, which has not been reported for microparticles. As a mechanism, complex formation of nanoparticles with lung lining layer proteins as such that this complex acts like a ferry boat for nanoparticles is conceivable. Microparticles would be excluded from this transport because of their larger size [70-72]. In addition, since microparticles are rapidly phagocytosed by lung surface macrophages, they are only shortly available for protein mediated transport. Surface modifications of nanoparticles are currently intensely studied in nanomedicine aiming at managing specific biokinetic behaviors of nanoparticles to distinctively target organs for diagnostic as well as therapeutic use [73]. As an example, drug delivery to the central nervous system via circulating nanoparticles requires surface modifications allowing receptor mediated nanoparticle translocation across the blood-brain barrier. Apolipoprotein-E coating of nanoparticles for LDL receptor mediated endocytosis in brain capillaries has been discussed [74-76]. Such highly desirable properties of nanoparticles must be carefully weighed against potential adverse cellular responses to targeted drug delivery by nanoparticles; a rigorous risk assessment is mandatory.

Nanoparticles not only offer a much larger surface area to be modified than microparticles of the same mass, but their larger number allows their dispersion into many more cells. For instance, a particle mass of 100 ng corresponds to only 2.4 × 10^4 ^particles (spheres of unit density) of 2 μm in diameter, but to 2.4 × 10^10 ^particles of 20 nm, or to 2.4 × 10^13 ^particles of 2 nm. Note that 20 nm particles comprise a major fraction of the number concentration of ambient aerosol particles [77] and that 2 to 10 nm particles are the primary particles originating from many combustion processes of which aggregated ambient ultrafine particles are made of.

Assuming that 100 ng of particles have accumulated in a secondary target organ like the heart or the brain, this would usually not be considered to be of any toxicological relevance for low toxicity particles. This is particularly true for the low particle number in the entire organ, when particles are 2 μm in diameter. However, if they are 2 nm particles, they exceed the number of cells in the organ easily by a factor of thousand (cell estimate is based on a 100 g organ and a cell volume of 4.2 × 10^-9 ^cm^3 ^corresponding to a cell of 20 μm in diameter). This enhances the probability of inducing adverse effects, although other factors like the subcellular localization of the nanoparticles, their chemistry and surface characteristics like surface proportional numbers of biochemically reactive centers are contributing as well.

There is plentiful literature on nanoparticle-cell interaction studies in vitro, however, the number of particles per cell was mostly not estimated but only the total mass added to the cell cultures is reported. In addition, rough estimates indicate that in most of these studies the nanoparticles to cell ratio was far beyond 1000:1, which largely exceeds any realistic dose in vivo. In many in vitro studies, particles are applied in doses of 1 - 100 μg per 10^5 ^cells. Assuming the application of the lowest dose of 1 μg particles per 10^5 ^cells and the use of spherical nanoparticles of unit density and a size of 50 nm, 2 × 10^5 ^nanoparticles per cell, or 2 × 10^10 ^nanoparticles per 10^5 ^cells, are applied. However, the effective dose per cell is very difficult to estimate, since these nanoparticles will not precipitate in the cell culture medium because of their minimal mass, but move by Brownian motion (diffusion), which is a non-directed motion. In addition, particle diffusion favours agglomeration to larger particle structures, which will then precipitate. By the time they do so, the particle characteristics, i.e. size distribution and concentration have obviously massively changed.

To estimate exposure conditions in vivo, we may assume an inhaled daily air volume of 15 m^3 ^for a healthy, adult, moderately active individual [39], a number concentration of ambient air particles of 3 × 10^4^/cm^3^, whereof 80 - 90% are nanoparticles [77], and a particle deposition fraction of 0.3 [39]. The first exposure estimate results in 1.4 × 10^11 ^nanoparticles being deposited per day or 6 × 10^9 ^particles deposited per hour. Even if particle number concentrations are higher, there is a maximal possible daily deposition of nanoparticles because of aerosol-physical limitations mostly due to diffusional coagulation. It is not possible to maintain the number concentration of nanoparticles aerosols above 1 × 10^6^/cm^3 ^for more than several minutes under normal ambient conditions. Hence, under such conditions maximally 2 × 10^13 ^nanoparticles per day or 6 × 10^11^/cm^3 ^per hour can be inhaled by an adult human. To estimate the ratio of nanoparticles per cell in vivo, we focus on the lung periphery, which is by far the largest compartment for nanoparticle deposition. We assume the alveolar surface of about 140^2 ^m in humans to consist of 2 × 10^10 ^epithelial type I cells and 3 × 10^10 ^epithelial type II cells, and to contain 6 × 10^9 ^alveolar macrophages [78]. Hence, for breathing of ambient aerosols, on average 6 nanoparticles will be daily deposited per cell in the alveolar region. Maximally, but not realistic, at the highest possible nanoparticles aerosol number concentration of 1 × 10^6^/cm^3^, an alveolar surface cell will receive on average 120 nanoparticles per hour. Even if we consider a maximum factor of 20 for inhomogeneous deposition beyond an otherwise rather homogeneous diffusional deposition in the peripheral lungs, the nanoparticle dose to some surface cells may increase by this factor at most.

For in vitro studies to become more relevant for assessing health effects by inhaled particles the nanoparticle dose per cell should reflect real exposure conditions [79].

#### Experimental studies on nanoparticle biokinetics

##### Human studies

Comprehensive biokinetics analysis of nanoparticles accumulation and retention in organs and tissues is not feasible in humans; because of ethical, but also technical reasons due to limiting resolution of existing imaging detection systems from outside the body or for body fluid samples.

So far, there is no evidence for a translocated nanoparticle mass fraction of more than 1% of the dose delivered to the lungs from reliable studies in humans [42,80-83]. However, there is indirect evidence of nanoparticle translocation in humans from recent inhalation studies in healthy subjects with diluted Diesel exhaust, which was found to impair the regulation of the vascular tone and fibrinolysis [84]. In addition, in a similar study, spontaneous alterations of electroencephalogram (EEG) signals in the frontal cortex were observed during and up to 1 h after exposure to Diesel exhaust [85]. From these studies it is not clear, whether the observed effects were initiated by translocated nanoparticles or by mediators released from the lungs in response to interactions with deposited nanoparticles.

Until now, histopathology revealed substantial particle loads in secondary target organs only after long term and massive exposure to microparticles; e.g. tar accumulation leading to increased blackening of lungs in smokers, particle or fiber accumulation in the liver and other organs of the reticulo-endothelial system in coal miners and asbestos workers [86,87]. Thereby, particle transport from the interstitium to the lymphatic system and further into the blood circulation was assumed. Similarly, in overload conditions translocation and accumulation of microparticles particularly in reticulo-endothelial organs were observed in experimental animals (dogs [88], rodents reviewed by [89]).

##### Animal studies

There is evidence for translocation of gold, silver, TiO_2_, polystyrene and carbon nanoparticles in the size range of 5 - 100 nm across the air-blood barrier from animal experiments. Either, nanoparticles were found in the blood circulation [31,90] and in secondary target organs [11-13,91-94], or thrombogenic effects were observed [95-97]. However, it still remains unclear, whether the translocated particle fractions exceeded 5% of the delivered lung dose (see also [41,98]). Recently, Chen and colleagues [99] reported an estimated translocated fraction of 1 - 2% for 50 and 200 nm polystyrene particles.

#### Quantitative assessment of nanoparticle translocation

Quantitative particle biokinetics as described in the Methods section allows the precise estimate of total and organ specific translocated nanoparticle fractions. Such data are currently available for:

- Inhaled iridium nanoparticles, 20 and 80 nm in diameter, in rats and mice [11-13]

- Inhaled carbon nanoparticles, 25 nm in diameter, spiked with radio-labeled primary iridium nanoparticles in rats [54]

- Instilled gold nanoparticles, 1.4 and 18 nm in diameter, in rats [94,100].

From the inhalation studies with iridium nanoparticles it became clear that such particles accumulate not only in secondary target organs but also in soft (connective) tissue and skeletal bone including bone marrow. In these studies, accumulation of 20 nm particles in all secondary target organs (liver, spleen, kidneys, heart, brain, reproductive organs) was in the range of 1 - 2% of the deposited dose at 24 h after administration. A similar fraction was found in the skeleton and up to 5% in the soft tissue. Hence, the total translocated fraction of nanoparticles reached just about 10% [54]. Furthermore, 20 nm iridium nanoparticles were poorly cleared from secondary target organs such that six months after a single one hour inhalation exposure, the total fraction of nanoparticles in all secondary target organs was still close to 0.1% of those initially deposited in the lungs, and all organs studied still contained nanoparticles [12,13]. Unfortunately, there are no further data on long term translocation of nanoparticles yet.

Even in the fetuses of pregnant rats in their third trimester, small but detectable fractions of translocated nanoparticles were registered [94].

#### Particle characteristics

##### Size

There is evidence from experimental studies that the translocation and accumulation of nanoparticles in secondary target organs depend on particle size; i.e. inhaled 80 nm iridium particles were shown to translocate about one order of magnitude less than the 20 nm ones, including accumulation in the skeleton and soft tissue [11,54]. Additionally, significant differences in the translocation and accumulation between 1.4 nm and 18 nm gold nanoparticles have been observed, with total translocated fractions of 8% and 0.2%, respectively, at 24 h after their intratracheal instillation [100]; both size categories were found in all secondary target organs investigated.

##### Material

Consequences of different materials on nanoparticle translocation and 24 h accumulation in secondary organs can be derived from inhalation studies with 20 nm iridium and 25 nm carbon nanoparticles. The fraction of carbon was significantly, 5 - 10 times lower than that of iridium (except in the liver) in any of the secondary target organs studied as well as in the skeleton or soft tissue. Note that both particle types are chain agglomerates made up of either 2 to 5 nm iridium or 5 to 10 nm carbon primary particles. Caution is required when these data are compared with those of 18 nm gold of similar size: there is not only the material difference, but gold nanoparticles are spherical and have a smooth surface. In addition, particle administration to the lungs was different (inhalation versus instillation). Yet, the total difference of almost 10% of translocated iridium and about 2% carbon chain agglomerates compared to 0.2% of the spherical gold nanoparticles within 24 h is striking, when considering particle material dependence only. There are more quantitative data required to better understand how particle materials influence biokinetics.

#### Dosimetry models for risk assessment

Experimental data help to develop and optimize mathematical models to predict the risk from inhaled particles. Kuempel and colleagues [101] showed reasonably good agreement between observed and predicted retained lung burden of poorly soluble fine and ultrafine particles in chronically exposed rats using dosimetry models. In addition, dosimetry models were further optimized to fit human data [102]. Thereby, the best fitting model was the one that included interstitionalization of particles, which is in accordance to the evidence obtained from experimental studies in humans. Such models can also be extended to include translocation of particles to secondary organs or differences in particle characteristics [103,104].

#### Summary remarks on nanoparticle translocation across the air-blood-barrier

Besides the discussed importance of size and material, other particle characteristics such as the surface charge (zeta potential) and surface structures are very likely to influence nanoparticle biokinetics. They determine the interactions of nanoparticles with proteins and cellular components and thereby the mechanisms for particle translocation and accumulation in extra-pulmonary organs. However, it needs to be emphasized that according to current knowledge, nanoparticles translocation and accumulation in extra-pulmonary organs is a minor clearance pathway for nanoparticles from the lungs compared to (long term) macrophage mediated nanoparticle clearance towards the larynx. Yet, while the latter pathway leads to particle excretion via the gastro-intestinal tract, nanoparticle translocation into the blood circulation systemically distributes nanoparticles and allows access to e.g. the cardio-vascular, the central-nervous and the reticulo-endothelial, i.e. the immune systems. Despite potential toxicological consequences for the organism when nanoparticles interact with these organ systems, it is still unknown whether it is the translocated nanoparticles that cause the epidemiologically established adverse effects. Particularly, it remains to be shown whether chronic exposure leads to sufficiently high nanoparticle doses to trigger or mediate responses leading to initiation and/or progression of disease. In addition, the release of mediators into the blood circulation needs thorough investigations: these mediators may be triggered or modulated by the well-known oxidative stress and pro-inflammatory responses to nanoparticles. Yet, even the importance of the dose metric is still debated. If nanoparticle mass is the effect determining metric, it appears very unlikely that sufficiently high doses in extra-pulmonary organs can be reached by inhalation. However, if nanoparticle number and (biochemically reactive) surface are the effect determining metrics, chronic exposure to nanoparticles may well be a health hazard; particularly, in susceptible individuals such as infants, the elderly and individuals with pre-existing cardiovascular and lung diseases.

Furthermore, the interaction of nanoparticles with the organism has to be studied at cellular and molecular levels, in lungs as well as in those secondary target organs which receive sufficiently high doses. Microscopic analyses of such organs from animal inhalation experiments may provide more detailed information about possible pathways responsible for (adverse) effects. It will be important to know which tissues, cells and/or subcellular compartments nanoparticles interact with and what particle properties are crucial for these interactions. Unrestricted crossing of the cellular membranes by nanoparticles facilitates not only their translocation into basically any organ but also into cells and subcellular compartment.

While unexpected nanoparticle access to secondary target organs at non negligible doses on a macroscopic scale as well as unexpected nanoparticle access to parenchymal and immune cells and to their subcellular structures like mitochondria and nuclei may result in adverse health effects, these interactions and pathways provide unforeseeable opportunities in the design of nanoparticles for diagnostic or therapeutic medical use in the new field of Nanomedicine.

## Abbreviations

AA-MS: atomic absorption mass spectroscopy; BAL: bronchoalveolar lavage; BALT: bronchus associated lymphoid tissue; EEG: electroencephalogram; EFTEM: energy filtering transmission electron microscopy; ESI: electron spectroscopic imaging; HRTM: human respiratory tract model; ICP-MS: inductively coupled plasma mass spectroscopy; ICRP: international commission of radiological protection; TEM: transmission electron microscopy; TiO_2_: titanium dioxide.

## Competing interests

The authors declare that they have no competing interests.

## Authors' contributions

MG was responsible for the design and realization of the studies at the microscopic level. WK was responsible for all inhalation studies and for the design and realization of the studies at the macroscopic level. Both authors prepared the manuscript and approved the final version.
